# Impact of climate change on the global circulation of West Nile virus and adaptation responses: a scoping review

**DOI:** 10.1186/s40249-024-01207-2

**Published:** 2024-05-24

**Authors:** Hao-Ran Wang, Tao Liu, Xiang Gao, Hong-Bin Wang, Jian-Hua Xiao

**Affiliations:** 1 Department of Veterinary Surgery, Northeast Agricultural University, Harbin, 150030 Heilongjiang People’s Republic of China; 2 Heilongjiang Key Laboratory for Laboratory Animals and Comparative Medicine, College of Veterinary Medicine, Northeast Agricultural University, Harbin, 150030 Heilongjiang People’s Republic of China

**Keywords:** West Nile virus, Climate change, Adaptation, Vector-borne pathogens

## Abstract

**Background:**

West Nile virus (WNV), the most widely distributed flavivirus causing encephalitis globally, is a vector-borne pathogen of global importance. The changing climate is poised to reshape the landscape of various infectious diseases, particularly vector-borne ones like WNV. Understanding the anticipated geographical and range shifts in disease transmission due to climate change, alongside effective adaptation strategies, is critical for mitigating future public health impacts. This scoping review aims to consolidate evidence on the impact of climate change on WNV and to identify a spectrum of applicable adaptation strategies.

**Main body:**

We systematically analyzed research articles from PubMed, Web of Science, Scopus, and EBSCOhost. Our criteria included English-language research articles published between 2007 and 2023, focusing on the impacts of climate change on WNV and related adaptation strategies. We extracted data concerning study objectives, populations, geographical focus, and specific findings. Literature was categorized into two primary themes: 1) climate-WNV associations, and 2) climate change impacts on WNV transmission, providing a clear understanding. Out of 2168 articles reviewed, 120 met our criteria. Most evidence originated from North America (59.2%) and Europe (28.3%), with a primary focus on human cases (31.7%). Studies on climate-WNV correlations (*n* = 83) highlighted temperature (67.5%) as a pivotal climate factor. In the analysis of climate change impacts on WNV (*n* = 37), most evidence suggested that climate change may affect the transmission and distribution of WNV, with the extent of the impact depending on local and regional conditions. Although few studies directly addressed the implementation of adaptation strategies for climate-induced disease transmission, the proposed strategies (*n* = 49) fell into six categories: 1) surveillance and monitoring (38.8%), 2) predictive modeling (18.4%), 3) cross-disciplinary collaboration (16.3%), 4) environmental management (12.2%), 5) public education (8.2%), and 6) health system readiness (6.1%). Additionally, we developed an accessible online platform to summarize the evidence on climate change impacts on WNV transmission (https://2xzl2o-neaop.shinyapps.io/WNVScopingReview/).

**Conclusions:**

This review reveals that climate change may affect the transmission and distribution of WNV, but the literature reflects only a small share of the global WNV dynamics. There is an urgent need for adaptive responses to anticipate and respond to the climate-driven spread of WNV. Nevertheless, studies focusing on these adaptation responses are sparse compared to those examining the impacts of climate change. Further research on the impacts of climate change and adaptation strategies for vector-borne diseases, along with more comprehensive evidence synthesis, is needed to inform effective policy responses tailored to local contexts.

**Supplementary Information:**

The online version contains supplementary material available at 10.1186/s40249-024-01207-2.

## Background

West Nile virus (WNV), the most widely distributed flavivirus globally, is a significant mosquito-borne virus [1]. It was first isolated in 1937 from the blood of a febrile woman in the West Nile region of Uganda. The earliest reported outbreaks occurred in the 1950s near Haifa, Israel [2]. Since the 1950s, WNV outbreaks have primarily occurred in Israel and various African countries [3, 4]. However, the epidemiology of WNV appears to have shifted since the 1990s due to the globalization of human trade and travel [1]. WNV was first detected in New York City in 1999 and subsequently spread rapidly throughout the entire Western Hemisphere, including the United States (US), Canada, and Argentina [5–7]. Concurrently, epidemic activity increased in Europe, the Middle East, and Russia [3, 4, 8]. In 2018, Europe experienced an unprecedented WNV epidemic, with human cases exceeding 1900, seven times higher than in previous seasons [9]. In 2020, locally transmitted human cases of WNV were reported for the first time in the Netherlands and Germany [10, 11]. Evidence suggests interactive WNV cycles on all continents except Antarctica [1].

The establishment of ongoing WNV transmission relies on the interactions among the virus, vectors, hosts, and environmental factors [12]. WNV can infect a wide range of vertebrate species, including most mammals, birds, and some reptiles and amphibians [13, 14]. Birds, serving as the primary amplifying hosts, play a crucial role in WNV proliferation. While humans and horses are susceptible to WNV, they are considered dead-end hosts [15]. In humans, WNV often results in asymptomatic or mild illness, but approximately 1 in 150 cases progress to neuroinvasive disease, potentially leading to encephalitis or death [16]. The primary vectors for WNV transmission are mosquitoes, particularly those belonging to the *Culex* genus. Mosquito bites are responsible for the vast majority of human WNV infections, although the virus can also spread through blood transfusions, organ transplantations, and potentially breastfeeding [17]. Given that WNV is transmitted by mosquitoes, its distribution depends on environmental conditions and is susceptible to the impacts of climate change [18]. For example, higher temperatures can accelerate viral replication, shorten the extrinsic incubation period in mosquitoes, promote vector abundance, enhance transmission efficiency, expand the suitability of vector habitats, and increase the probability of avian migration across regions [19, 20]. Additionally, precipitation patterns have a significant impact on mosquito breeding and abundance, thus affecting the spread and geographical distribution of WNV [18].

The current body of evidence strongly indicates that climate change directly impacts the spread and proliferation of vector-borne illnesses, including WNV [21]. Numerous studies have demonstrated that areas vulnerable to WNV transmission could expand or shift due to climate elements. This encompasses projecting future global climate change scenarios, examining how vector species respond to environmental shifts in laboratory settings, and conducting field research in regions where outbreaks occur. There is some evidence of WNV emerging or re-emerging in high-latitude regions and at the edges of current endemic zones [22–26]. For example, in North America, the suitable range for WNV is projected to extend northward and to higher altitudes by 2050 and 2080, potentially leading to new infections in both native and non-native species [22]. In Europe, increased WNV cases and new outbreak locations are predicted under future climate scenarios, especially at the margin of current transmission areas [23]. In South America, high risk areas for WNV might shift between 2046–2065 and 2081–2100, with more pronounced changes under high greenhouse gas emission scenarios, potentially altering the current WNV distributions in some countries (e.g., parts of Bolivia, Paraguay, and Brazil) [24]. Moreover, existing surveillance data support the overall trend of heightened WNV risk due to climate change. For instance, in the Powder River Basin of Montana and Wyoming, US, the WNV mortality rate in the wild bird population was significantly higher in 2003 (the sixth most sweltering summer historically) than in 2004 and 2005 [25]. In Germany, the extreme heat in the summer of 2018 (the second most sweltering and desiccated summer historically) theoretically played a pivotal role in reducing the average extrinsic incubation period in mosquitoes, resulting in rapid viral amplification and increased transmission risks to vertebrate hosts [26]. However, the impact of climate change on WNV distribution may vary geographically, and some areas may see a decrease in cases. For example, while Keyel et al. predicted a general increase in WNV cases in 2021, a subsequent study indicated that future cases may decrease in areas outside the boundaries of the original study area in New York [27, 28].

While efforts to mitigate climate change are essential to reduce CO_2_ emissions and lessen potential future impacts, there is an increasing need to focus on adaptation strategies as well. These include various short-term measures at different levels to address the immediate effects of climate change [29]. Adaptation approaches aim to enhance resilience in health systems, preparing them to manage and minimize the health consequences of climate change [29]. Given the commitments countries have made to the Paris Agreement and Sustainable Development Goals, along with the growing global evidence base for climate change's impact on disease spread, nations have begun developing and implementing policy responses as components of national climate adaptation plans [30]. Insights into the expected magnitude of climate change impacts on WNV and associated adaptive responses can help inform best practices to mitigate public health impacts from the climate-induced spread of disease.

Contemporary prioritization in Canada of investigative pursuits on emerging human and animal diseases under climate change scenarios indicated that WNV is a disease requiring primary attention [31, 32]. Since the Intergovernmental Panel on Climate Change (IPCC) Fourth Assessment Report in 2007, the health impacts of climate change have garnered significant research focus [33]. This attention has increased further following the Fifth Assessment Report in 2014 and the 2015 Lancet Commission on Climate Change and Health, leading to a growth in the number of related publications [34, 35]. In addition to highlighting the impacts of climate change, these articles also emphasize to some degree specific interventions or policy responses within defined countries and regions. To our knowledge, a comprehensive review of the global impacts and adaptation responses related to climate change and WNV has not been conducted. Such a review is necessary to consolidate existing evidence, explore how climate change influences the spread of WNV, and identify the most effective strategies for developing adaptation policies.

In summary, this scoping review aims to address two core questions:

i) What types of evidence exist regarding the impact of climate change on the global transmission of WNV?
ii) What adaptation measures have been proposed or implemented in response to climate change?

Our primary focus is to elucidate the climatic drivers of WNV to better inform these strategies. This approach is intended to serve as a foundation for future research that may delve into comprehensive public health policies and adaptation measures.

## Methods

### Protocol and registration

We used a scoping review methodology to select studies for inclusion in this synthesis. Our review followed an established protocol, guided by the PRISMA Scoping Review Extension (PRISMA-SCR) and published scoping review methodology [36–38]. It was registered with the OSF Registries (https://osf.io/9j2as) on December 25, 2023, to ensure transparency [39, 40].

### Search strategy

We conducted systematic searches across four databases—PubMed (MEDLINE), Web of Science, Scopus, and EBSCOhost—to identify relevant peer-reviewed publications on climate change and WNV between January 2007 and December 2023 without imposing language restrictions. Our literature searches employed terminology related to climate change and the diseases of interest. Terms for climate change were taken from the search strategy used in Sweileh’s (2020) bibliometric analysis of climate change and health publications: “climat* Change” OR “global warming” OR “changing climate” OR “climate variability” OR “greenhouse gas” OR “rising temperature” OR “extreme weather” OR “greenhouse effect” [41]. Disease-specific terms included were: “West Nile virus” OR “WNV host” OR “WNV vector”. Full search strategies for each database are provided in supplementary materials (Additional file 1).

This search strategy was designed to comprehensively capture all original studies examining the associations between meteorological, climatological, ecological, or environmental change factors and the transmission dynamics, outbreaks, risks, or adaptations of WNV. By conducting systematic searches across key databases, supplemented by targeted topic strings, our strategy ensures reproducibility and effectively summarizes contemporary evidence illuminating the connections between WNV and climate amidst escalating changes.

### Eligibility criteria

The criteria for including and excluding articles in our analysis are outlined in Table 1. We examined literature since 2007 to capture research conducted after the IPCC Fourth Assessment Report’s release, representing a milestone driving expanded climate-health investigations [33, 41]. Focusing on this period enhances relevance and rigor by concentrating on studies consciously examining climate-related impacts during intensifying change. Further augmenting stringency, we concentrated solely on original quantitative and qualitative investigations published in English-language peer-reviewed academic journals. Together these boundaries help systematically extract recent high-quality evidence elucidating shifting WNV transmission dynamics amidst climate change while delineating adaptations instituted since an authoritative global assessment.

**Table 1:** Criteria for inclusion and exclusion

Category	Inclusion	Exclusion
Concept	Articles on climate or climate change impacts on disease emergence, transmission or spread Interventions or climate change adaptation measures related to disease emergence, transmission or spread Articles on West Nile virus	Articles on disease prevention and control interventions f or West Nile virus that are not explicitly related to climate change Articles on climate impacts or interventions related to mosquito species with no mention of implications for WNV emergence, transmission or spread
Type of evidence sources	Original research studies	Review, thesis, conference, etc
Language	Articles in English	Other languages
Timeframe	Articles published between 2007 and 2023	Articles published prior to 2007
Publication status	Articles published or in press	Pre-print articles

### Screening and study selection

We used the systematic review software NoteExpress 3.8.0.9455 (Beijing Aegean Sea Software Company, Beijing, China) to implement standardized screening and selection procedures. Two independent reviewers carried out an initial screening of titles and abstracts to filter articles that met basic eligibility criteria, with a third reviewer resolving any discrepancies. Subsequently, these two reviewers conducted full-text evaluations of the retained articles to ensure compliance with all inclusion criteria as outlined in the predefined protocol. Any disputes again triggered third-reviewer arbitration to achieve consensus.

### Data extraction

We used a predefined covidence data extraction framework to systematically characterize key article features including 1) identifiers like title, author(s), and year; 2) specific objectives, study populations, WNV research priority (primary/secondary), and geographic focus; and 3) findings of the paper, such as nature of the evidence for climate change impacts on disease emergence, transmission or spread and/or policy responses, interventions or adaptations [42, 43].

We categorized the geographic focus of articles into six regions: North America, South America, Europe, Africa, Asia, and Oceania, with multi-regional studies classified as global. The study populations analyzed included humans, mosquitoes, birds, and horses. Investigations encompassing more than one species were labeled as ‘multiple species’, and studies that did not specify their focus were marked as ‘unspecified’. The central disease under investigation in all articles was WNV. Articles primarily focused on WNV dynamics were categorized under ‘primary’ interest level, while those analyzing WNV in conjunction with other vector-borne diseases were deemed of ‘secondary’ interest.

The findings of the paper regarding evidence or arguments presented on the impacts of climate change (including extreme weather, rising temperatures, and/or climate variability) on WNV emergence, transmission, or spread were recorded. To clearly understand the impacts of climate change on WNV, articles were grouped into two main categories: 1) climate-WNV associations, and 2) climate change impacts on WNV, as categorized by Kulkarni et al. in their study of the impact of climate change on global malaria and dengue fever [38]. The articles defined as climate-WNV associations mainly refer to the impacts of climatic and seasonal factors (e.g. temperature, precipitation, and seasonal variations) on WNV transmission and spread within a certain time frame. Articles defined as climate change impacts on WNV are further categorized into two types: those with clear evidence of climate change or climate anomalies during the study period affecting WNV transmission and spread, and those with projections of future WNV transmission and spread under climate change scenarios.

The findings of the paper pertaining to evidence for policy responses, interventions, or adaptive measures addressing the impacts of climate change on disease emergence, transmission, or spread were documented. Specifically, the nature of the evidence or arguments presented regarding policy measures, interventions, and/or adaptations to mitigate the effects of climate change on the emergence, propagation, or spread of WNV were recorded. The United Nations Environmental Program (UNEP) handbook on methodologies for assessing climate change impacts and adaptation strategies outlines a typology of adaptation measures to safeguard human health from climate change [44]. These encompassed five categories of measures: (1) surveillance and monitoring, (2) infrastructure development, (3) public education, (4) technology or engineering strategies, and (5) medical interventions. The content of the article on adaptation strategies is categorized according to the UNEP manual and in the context of the WNV case.

### Quality assessment of included literature

The quality of the included articles was assessed using the Joanna Briggs Institute Prevalence Critical Appraisal Tool [45]. All selected studies were scored using the 10 quality control items suggested by the tool. A score of one was awarded for each item fulfilled while a zero score was awarded for each unmet item. Score aggregates were generated and studies were classified as either low (0–3), moderate (4–6), or high (7–10) quality.

### Web development

Most reviews traditionally present evidence in a tabular format, which consumes a considerable portion of the article’s space and often hinders easy navigation through the key information [36, 38]. In this study, we used the R Shiny interactive web application framework to develop an online-accessible website that presents evidence on the impact of climate change on WNV transmission and dissemination [46]. This website allows visitors to query and download information on the effects of climate change on WNV transmission and spread at any time and from any location. This method provides a novel way to access and understand the synthesized evidence in a clearer and more convenient manner.

## Results

### Characteristics of included studies

**Fig. 1:**
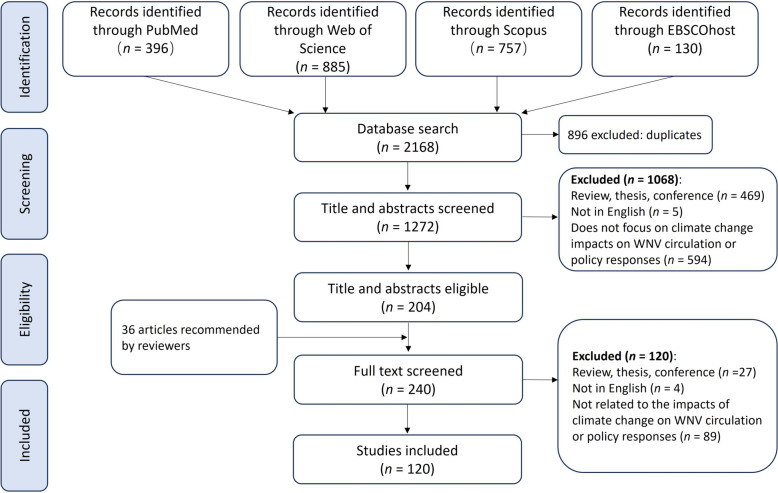
Flowchart diagram illustrating the article search and selection process

Initially, 2168 articles were retrieved from four databases: Web of Science, PubMed, Scopus, and EBSCOhost. After removing 896 duplicates, 1272 articles remained (Fig. 1). Following title and abstract screening, 1068 articles were excluded as irrelevant, leaving 204 for full-text review. This resulted in 105 articles meeting inclusion criteria, focusing on the association between climate/weather and WNV or its transmission due to climate changes.

To comprehensively cover literature on the impact of climate change on WNV, we used specific search terms based on key themes from prior studies [37, 38]. Although these terms helped in retrieving targeted and relevant literature, their specificity might have restricted the scope, possibly excluding significant studies that broader terms could have included. Hence, the reviewers recommended 36 relevant articles, which we screened and retained 15 articles according to the inclusion criteria.

The comprehensive review included 120 studies divided into two categories: 83 studies focused on the associations between climate/weather and WNV, and 37 studies examined the impacts of climate change on WNV transmission. All the reviewed evidence and related adaptation responses are available for exploration and download through a dedicated Shiny web application (https://2xzl2o-neaop.shinyapps.io/WNVScopingReview/).

### Publication year

**Fig. 2:**
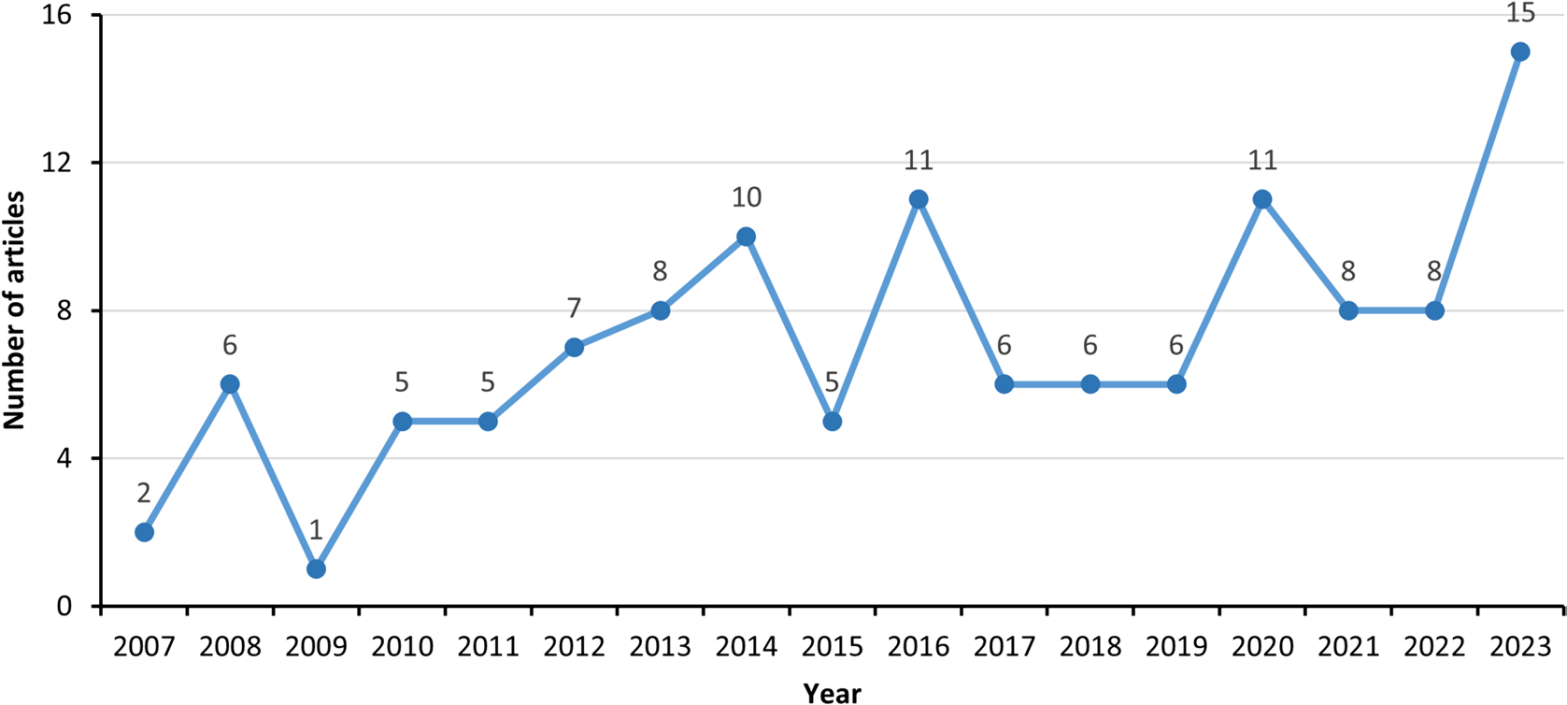
Distribution of the publication years in all articles included from 2007 to 2023

The number of published studies on climate change and WNV has increased over time, with a sharp rise observed after 2013 (Fig. 2). Regarding the temporal distribution of relevant literature, two key observations can be made.

First, only 26 articles were published between 2007 and 2012, of which 21 articles focused on the associations between climate/weather [47–67] and 5 articles examined the impacts of climate change on WNV [25, 68–71]. The earliest study on climate/weather factors and WNV, published in 2007, analyzed the association between precipitation and human WNV incidence in the US during 2002–2004. The first article on the impacts of climate change on WNV, published in 2007, investigated WNV prevalence in wild Greater Sage-Grouse populations across Montana and Wyoming during 2003–2005. The relatively small number of studies before 2013 indicates that relevant research was still in its infancy stage.

Second, most studies on this topic (*n* = 94) emerged after 2013, corresponding to the release of the IPCC Fifth Assessment Report in 2014 and the Lancet Commission on Climate and Health in 2015 [34, 35]. As authoritative reviews synthesizing the state-of-the-art science on anthropogenic climate change and its health consequences, these landmark reports have stimulated new research assessing climate impacts on infectious diseases like WNV.

### Study location

**Fig. 3:**
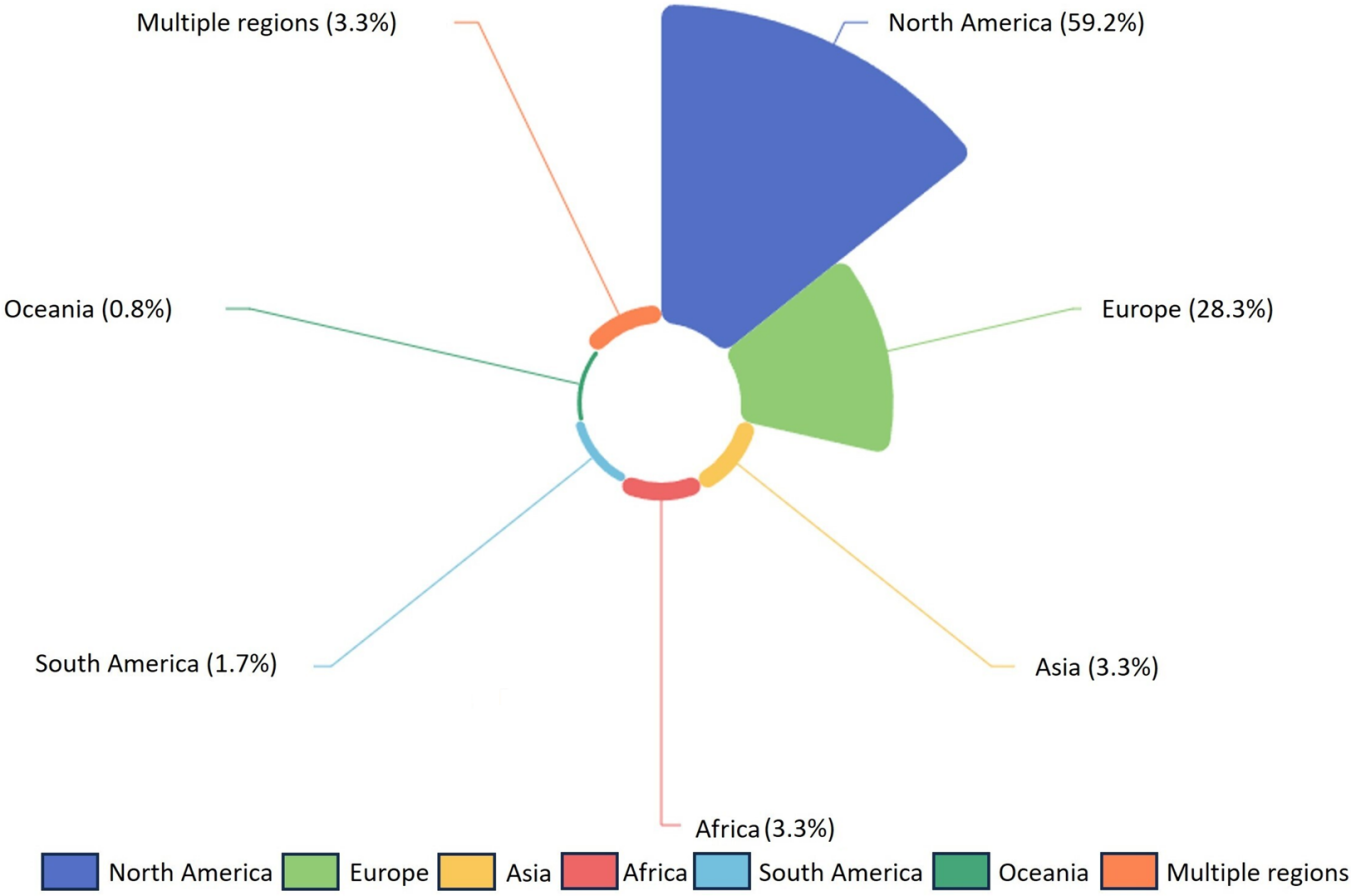
Geographical distribution of the study areas in all articles included from 2007 to 2023

The geographical distribution of study locations examined in the articles is shown in Fig. 3. The most frequently studied region was North America, representing 59.2% of articles (*n* = 71). Within North America, 53 articles focused on the US [25, 27, 28, 47–62, 68, 72–104], 17 on Canada [31, 32, 63, 64, 69, 105–116], and 1 covered the entire continent [22]. Europe was the second most studied region, accounting for 28.3% of articles (*n* = 34) [23, 26, 65, 70, 71, 117–145]. The other world regions assessed were Asia (*n* = 4; 3.3%) [66, 146–148], Africa (*n* = 4; 3.3%) [67, 149–151], South America (*n* = 2; 1.7%) [24, 152], and Oceania (*n* = 1; 0.8%) [153]. Only 4 articles (3.3%) [51, 154–156] included multiple global regions and were classified as the “global” studies.

Research on WNV has focused on two regions, North America and Europe, which corresponds to the high incidence and disease burden from epidemics reported in these two regions over the past two decades. In the US, between 2007 and 2022, there were 32,600 confirmed or suspected human WNV cases reported to the Centers for Disease Control and Prevention, particularly concentrated in California, Colorado, and Texas [157]. WNV remains the leading cause of mosquito-borne disease in the US, accounting for 83.0% of the reported cases in 2020 [158]. In Canada, since the virus's emergence in 2001, there have been over 5000 lab-confirmed human cases, with around 20.0% of patients experiencing neurological complications [159, 160]. Additionally, it is estimated that up to 27,000 cases may have gone unreported, given the largely asymptomatic nature of WNV infection [160]. Similarly severe WNV outbreaks have hit Europe in recent years — its 2018 epidemic exceeded 1900 confirmed human cases, surpassing all previous years in scale and distribution [161]. The heavy health and economic toll has reasonably triggered intensive research interests in examining environmental risk factors such as climate change. Study interests and public health priorities understandably tend to align with acute epidemic events and tangible disease burden.

Research on WNV in regions like Asia, Africa, South America, and Oceania has been comparatively sparse. This imbalance may stem from various factors, such as a lower prioritization due to limited epidemiological data and clinical cases, often attributed to suboptimal surveillance systems. Additionally, the allocation of public health resources in these regions might be challenged by competing health issues, alongside barriers to conducting coordinated multi-national research. For example, in South America, inconsistencies between actual and reported WNV cases arise from symptomatic similarity with other arboviruses and limitations in differential laboratory diagnostics [24]. Moreover, mild and self-resolving cases may remain undocumented. Meanwhile, more severe cases can also be under-diagnosed, owing to a lack of accessible healthcare facilities and logistical constraints on sample transportation and testing [12].

Regional differences in climate, vector ecology, and host community characteristics contribute to variations in WNV transmission patterns and health impacts. For example, the primary vectors of WNV display distinct seasonality under varying climatic conditions [68]. Furthermore, viral strains may evolve different levels of pathogenicity in diverse host species and environmental settings [84]. Consequently, collaborative multi-regional research is essential to formulate prevention policies that are specifically tailored to different regions. Additionally, integrating knowledge and assessment tools is crucial to further understand the environmental and social factors driving WNV transmission.

### Study population

**Fig. 4:**
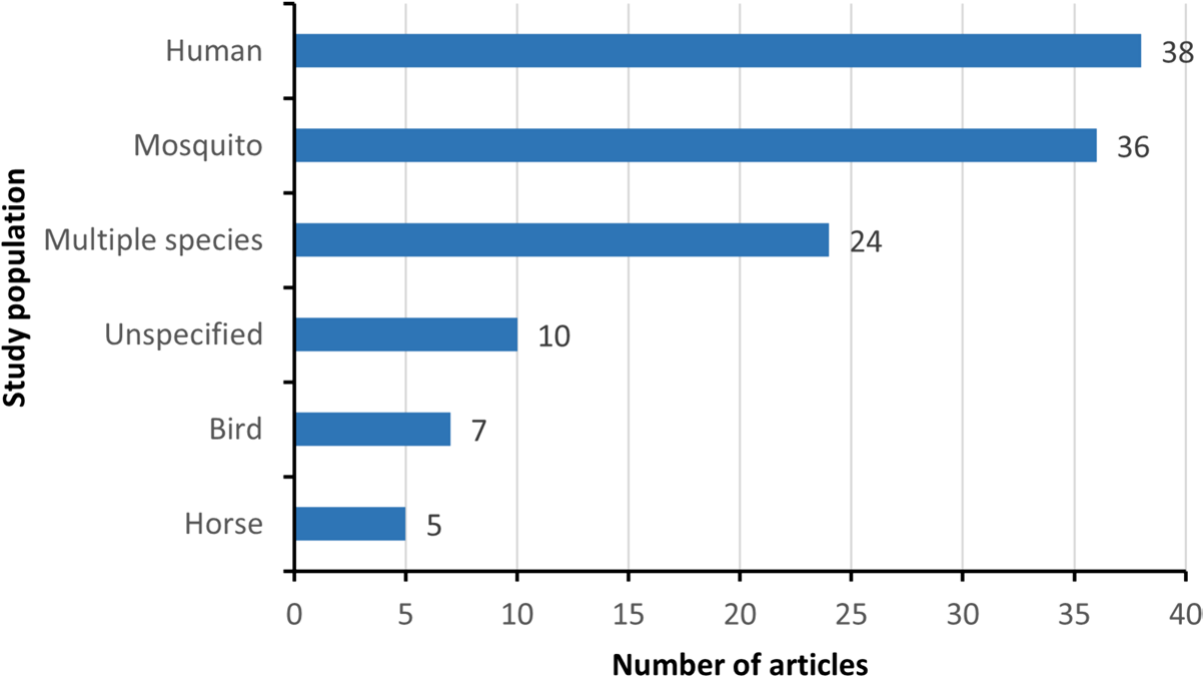
Distribution of the study populations in all articles included from 2007 to 2023

The majority of research articles (*n* = 103; 85.8%) focused exclusively on WNV, its vectors, or hosts. The remaining 17 articles (14.2%) examined WNV in conjunction with other mosquito-borne diseases, such as dengue fever and Rift Valley fever. The most studied subject was human WNV cases (Fig. 4), examined in 31.7% of articles (*n* = 38) [23, 27, 47, 48, 52–54, 56, 58, 59, 62, 66, 71, 73, 78, 82, 83, 90, 96, 98, 102, 109, 118, 122, 124–126, 128, 130–135, 140, 143, 145, 148]. Mosquito vectors[49, 55, 57, 61, 65, 68, 69, 72, 74, 80, 84, 87, 88, 91–93, 95, 97, 101, 103, 105, 107, 108, 110, 111, 116, 121, 127, 129, 136, 139, 144, 150, 152, 153, 155] and multi-species [22, 26, 28, 51, 60, 64, 67, 70, 75–77, 79, 81, 86, 94, 99, 100, 106, 117, 119, 123, 141, 142, 146] were investigated in 36 (30.0%) and 24 (20.0%) studies, respectively. A smaller percentage of articles (*n* = 10, 8.3%) failed to specify the study population [24, 31, 32, 51, 112, 113, 120, 151, 154, 156]. Limited studies focused solely on bird hosts (*n* = 7, 5.8%) [25, 50, 89, 104, 114, 115, 138] or equine hosts (*n* = 5, 4.2%) [63, 85, 137, 147, 149].

The majority of WNV research has focused on human infection. It's estimated that about 1 in 150 infected individuals develop a severe, long-lasting illness [162]. High incidence rates in humans have been linked to environmental factors such as extensive irrigated croplands and rural settings [54]. Mosquito vectors, particularly *Culex* species, play a crucial role in WNV transmission cycles, with their abundance influenced by factors like the urban heat island effect, the presence of water bodies, and the extent of irrigated farmland [54, 129].

However, there is a significant gap in the number of animal-focused studies compared to human studies. In North America, over 28,000 equine cases of WNV have been reported since 1999 [163]. Additionally, in the US alone, the virus has impacted over 300 bird species, with estimated deaths in the millions [164]. Juvenile dispersing birds have been demonstrated to play a vital role in the long-distance dispersal and rapid spatial spread of introduced WNV strains across North America [165]. Given the importance of the role of animals in the transmission and evolution of WNV, there is a need to strengthen research on the impacts of climate change on the transmission and spread of WNV in animals.

### Climate-WNV associations

Among the 83 articles examining climate/weather associations with WNV, temperature was the most studied factor (*n* = 56, 67.5%) [28, 47, 49, 51, 52, 54, 55, 58–61, 63, 64, 66, 72, 75, 76, 79–82, 84, 86–89, 92–94, 98, 105, 107–110, 117–130, 132, 134, 137–139, 147, 150]. All these studies showed increased WNV transmission probabilities or cases within certain temperature ranges. Precipitation was assessed in 34 studies (41.0%), with 13 showing a positive correlation, 13 indicating a negative correlation, 7 revealing mixed positive/negative correlations, and 1 indicating no correlation with WNV risk [28, 47, 48, 52–55, 58, 63, 67, 72, 77, 79, 81, 82, 86–88, 92, 93, 98, 105, 109, 110, 125, 126, 128, 131, 136, 137, 139, 146, 149, 150]. Drought events and warmer winters were investigated less frequently, in 8 (9.7%) [28, 56, 72, 74, 85, 119, 126, 146] and 5 (6.0%) [50, 65, 83, 92, 106] studies, respectively. Four articles (4.8%) showed a correlation between humidity and WNV risk, with 3 [47, 80, 91] showing a positive correlation and 1 [119] showing a negative correlation. Nine studies (10.8%) found links between WNV activity and climate-driven seasonal shifts [57, 61, 62, 73, 78, 90, 95, 96, 133], while 2 (2.4%) reported increased transmission associated with flooding events [135, 153]. Three studies (3.6%) reported the correlation between WNV risk and winds/hurricanes [91, 97, 119].

#### Temperature and WNV

Ambient temperature is a critical driver influencing WNV transmission through direct and indirect impacts on vectors and hosts [49, 71]. Specifically, higher temperatures accelerate viral replication and shorten the incubation period in mosquitoes, fuel vector population growth, increase transmission efficiency, and expand vector habitat suitability [49]. In Israel, positive temperature anomalies were linked to greater mosquito abundance and ensuing human cases [66]. Similarly in Canada, higher mean temperatures are associated with increased *Culex* populations and elevated WNV infections [129]. Moreover, there is a trend towards increased risk around large metropolitan areas characterized by urban heat islands, for example in the United Kingdom [129]. Phenomena such as warm winters and hot summers due to increased temperatures have also contributed to the rise in WNV infection rates [50, 65, 83, 106]. The mean temperature is a strong predictor of the presence of WNV in *Culex* mosquitoes, and this relationship is unimodal [76]. The optimal temperature range for WNV transmission is identified as 22.7–30.2 °C [75]. Outside this range, particularly at temperatures below 17.0 °C, vector competence significantly declines, reaching a relative risk near zero [76]. It is important to note that this lower temperature threshold can vary among different vector species. Moreover, extreme heat events may further amplify outbreak magnitude [65, 66]. However, it is important to note that an increase in temperature does not necessarily mean an increase in disease incidence altogether. For example, temperatures above 30 °C reduced survival of *Culex tarsalis* and slowed the growth of WNV in *Culex* mosquito [166].

In addition, ambient temperature rise under climate change may indirectly alter WNV transmission by shifting bird host ecology and associated vector exposures. Models project warming could expand bird infection prevalence to higher latitudes as longer activity seasons enable more transmission events [114]. While temperature alone allows increased vector habitat suitability and viral replication at mid-range optima, cascading impacts on avian immunity, migration timing, vector-host overlaps, and habitat ranges could potentially override direct effects. For instance, warmer climates have prompted earlier nesting in British birds, potentially leading to offspring hatching during peak mosquito seasons, increasing young birds’ exposure to vectors [167]. This phenomenon is exemplified in Caillouët et al.’s study, which demonstrates how the end of the nesting season aligns with higher mosquito populations, potentially escalating WNV transmission risks during these periods [168].

#### Precipitation and WNV

The positive correlation between elevated rainfall pre-outbreak and intensified WNV vector abundance/infection has been well documented [47, 125, 126]. For example, a 10 cm rise in summer precipitation was associated with 0.39 more WNV-positive *Culex* mosquitoes per 1000 tested in South Africa [67]. In the US, every 1 cm precipitation increase was linked to a 15% greater WNV incidence [86]. In Australia and Srpska, flooding due to extreme precipitation events creates favorable conditions for WNV transmission, as waterlogged environments can support larger populations of waterbirds and mosquitoes, increasing the likelihood of virus spread [135, 153]. However, precipitation effects on WNV vary across regions and timescales, likely due to place-based differences in viral strain, vector, and host ecology. For example, a negative correlation between total monthly precipitation and the number of WNV cases was observed in Europe [128]. Similarly, in years of increased human WNV incidence in Israel, there was a significant decrease in spring precipitation [146]. In America, extreme drought caused by extremely low precipitation is a potential amplifier of WNV virus transmission and can further increase the risk of WNV transmission [56, 85]. The cause of this phenomenon may be related to the fact that below-average precipitation creates limited water resources for mosquitoes, thereby increasing close contact between hosts and infected mosquitoes at remaining water sources [169]. In addition, both positive and negative correlations of precipitation on WNV incidence have been observed in the eastern and western parts of the US at different time scales [53, 82].

#### Humidity, wind speed and WNV

Humidity and wind speed play important and complex roles in WNV transmission dynamics, but the impacts vary widely across ecosystems. For instance, higher humidity increased the probability of human infection with WNV in the US [47], and positive correlations were found between soil moisture and vector indices [80]. However, a Greek study conversely found negative relative humidity-WNV case correlations [119]. A study in New York and Connecticut showed an inverse U-shaped relationship between soil moisture and WNV-infected mosquitoes, with high infection associated with drought, but also an increase associated with wetter conditions—both patterns can be present at the same time [27]. Meanwhile, wind may impact disease transmission by influencing mosquito movement. For example, low wind speeds were found to be associated with the capture of WNV-infected mosquitoes during the same week that human cases of WNV emerged in Greece [119]. This may be related to the fact that high wind speeds reduce the chances of a mosquito blood meal, thus reducing the chances of human WNV cases [119]. Additional hypotheses, including storm roles in bird migration contributing to WNV transmission [170], require further investigation.

#### Climate-driven seasonal shifts and WNV

Climate-driven seasonal shifts are also important factors influencing WNV spread and outbreak magnitudes. For example, Texan counties experience major spikes following wet springs and hot, dry summers [73]. In Suffolk County, warm and dry conditions in early spring have been shown to increase WNV infection in *Culex* mosquitoes [74]. Patterns of dry, hot temperatures following wet years also increase WNV infections [78]. Broader European analyses suggest that anomalous seasonal temperatures and dry winters exacerbate seasonal amplification and drive WNV outbreaks [133]. These climate-mediated seasonal effects likely arise through multiple mechanisms affecting vector reproduction, host immunity, viral replication rates, and transmission efficiency at different phases [170]. As climate change intensifies precipitation variability and seasonal temperature extremes, such seasonal shift tipping points may become more frequent. Therefore, improved surveillance programs that are responsive to emerging seasonal shifts remain essential for predicting and mitigating transmission at fine geographic and temporal scales.

#### Summary

While climatic factors have a significant impact on the spread and transmission of WNV, many other factors also influence the complexity of the transmission dynamics. Land use, global trade, bird migration patterns, landscape features, and socioeconomics also partially determine the geographic distribution of infections [50, 80, 86]. For example, areas with older infrastructure, lower incomes, high percentages of cropland, and large rural populations have more landscape features and environmental conditions favorable to vector habitat, which increases local WNV risk [54, 80]. Therefore, operationalizing the “One Health” paradigm through collaborative surveillance, modeling, and mitigation across veterinary, human, wildlife and environmental health remains imperative for fully anticipating and responding to shifting WNV.

### Climate change impacts on WNV

Among the 37 articles examining climate change impacts on WNV, the majority (*n* = 28; 75.7%) predicted the impact of climate change on WNV [22–24, 27, 31, 32, 69, 70, 99–104, 111–116, 141–143, 145, 151, 152, 155, 156]. Specifically, high latitude regions, areas with immunocompromised populations, locations prone to extreme weather events, and marginalized communities were expected to be more affected [22, 24, 103]. Additionally, 8 articles (21.6%) provided substantial evidence that climatic variability phenomena have already affected the transmission and distribution of WNV during recent outbreaks [25, 26, 68, 71, 140, 144, 148, 154]. Only 1 article (2.7%) focused on developing a national indicator framework for monitoring climate change impacts on infectious diseases [51].

#### Evidence of future climate change impacts on WNV

In the review, most evidence predicts that future climate change may affect the spread and distribution of WNV [22–24, 111, 114, 151, 155, 156]. In North America, the projected climatic suitability range for WNV in 2050 and 2080 is expected to expand northward and into high-altitude areas, potentially leading to infections in novel and native hosts [22]. In Europe, studies project heightened WNV infection rates and new endemic areas under future climate scenarios, particularly at the margin of current transmission zones (e.g., eastern Croatia, northeastern and northwestern Turkey) [23]. Notably, recent evidence also confirms local transmissions as far north as Germany and the Netherlands, indicating an expansion of risk areas beyond those previously identified [10, 11]. In South America, high-risk areas for WNV may shift between 2046–2065 and 2081–2100, becoming more pronounced under high greenhouse gas emission scenarios, potentially altering the current WNV distribution in some countries (e.g. parts of Bolivia, Paraguay, and Brazil) [24]. In Morocco, the suitable habitat range for *Cx. pipiens* is projected to expand into new central and southeastern areas by 2050, increasing the risk of WNV transmission [151].

#### Current evidence of climate change impacts on WNV

In addition to predictive studies on the future, existing evidence also demonstrates that climatic variability phenomena have already affected the transmission and distribution of WNV in some regions [25, 26, 144, 148]. In the Powder River Basin of Montana and Wyoming in the US, WNV-related mortality rates in bird populations were significantly higher in 2003, the sixth warmest summer on record, than in 2004 and 2005, the 86th and 41st warmest, respectively [25]. Although this increase in mortality coincided with higher temperatures, it is crucial to consider that 2003 also marked a period of the virus’s initial introduction into the region. This introduction likely contributed significantly to the observed mortality rates, as populations are often most vulnerable when a pathogen first emerges. In Germany, the extreme heat of the summer of 2018 (the second hottest and most arid summer on record locally) was speculated to be an important reason for the decreased mean extrinsic incubation period values in mosquitoes, leading to rapid viral amplification and increased risk of transmission to vertebrate hosts [26]. Additionally, the detection of WNV-infected *Uranotaenia unguiculata* in northern Germany in 2016 presents another case of climate change driving the northward spread of mosquito species and WNV [144]. In Israel, an intense heat wave and a spike in summer temperatures were observed during WNV outbreaks [148].

#### Summary

The extent of climate change impacts on WNV transmission depends on local regional conditions, including population immunity levels and vector abundance [99, 101, 103]. In areas where comprehensive vaccination programs for animals susceptible to WNV, such as horses, are in place, alongside robust public health infrastructure and strong vector monitoring and control systems, the impact of WNV may be significantly mitigated or even negligible [103]. For example, predictions for the island scrub-jay in California showed that vaccinating ≥ 60 individuals during WNV outbreaks could decrease the risk from ≥ 22% to ≤ 5% [104]. Undoubtedly, strengthening broad-spectrum socioecological resilience through surveillance, preparedness, vector management, and medical capacity building remains paramount for sustainable health amidst climate and global change [101]. However, these anthropogenic measures require considerable regional coordination and resource mobilization, frequently lacking in disproportionately impacted communities. Therefore, actualizing equitable and adaptive WNV resilience necessitates comprehensively integrating climatological, environmental, veterinary, wildlife, genetic, immunological, and public health data into prediction frameworks and response protocols prioritizing vulnerable populations. International organizations must lead in facilitating such collaborative resilience measures globally.

### Adaptation strategies to address climate-driven WNV transmission and spread

**Fig. 5:**
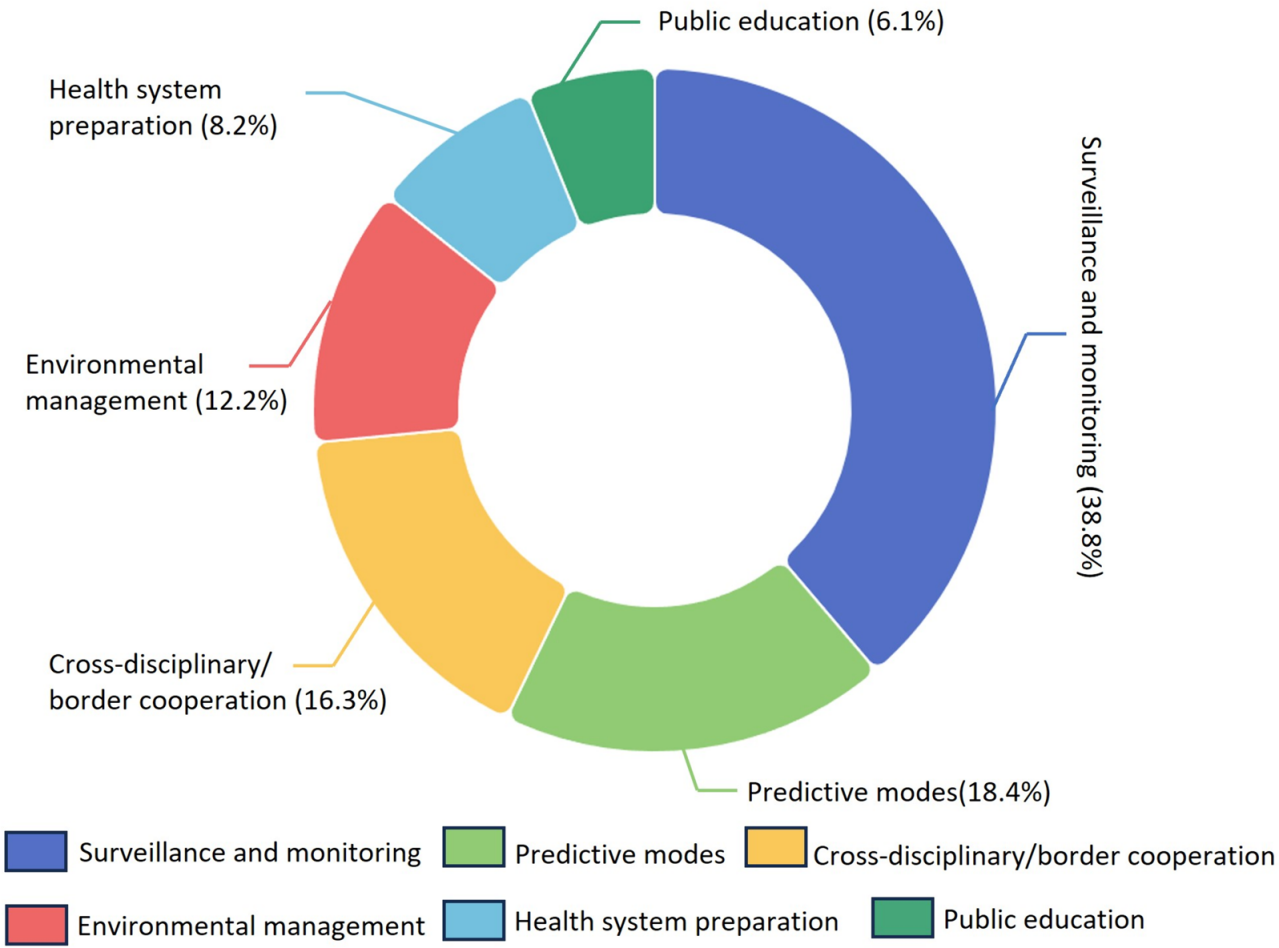
Classification of 49 articles that proposed or discussed adaptive strategies against West Nile virus risks in response to climate change, divided into six categories based on United Nations Environmental Program criteria

Among all 120 reviewed articles, 49 proposed or discussed adaptive strategies against WNV risks in response to climate change. These measures were categorized into six groups based on UNEP criteria and the case of WNV (Fig. 5) [44]: surveillance and monitoring (*n* = 19; 38.8%) [22, 23, 56, 65, 69, 75, 85, 92, 99–101, 106, 117, 120, 123, 135, 148, 151, 152]; predictive models (*n* = 9; 18.4%) [49, 70, 74, 81, 84, 98, 103, 105, 111]; cross-disciplinary/border cooperation (*n* = 8; 16.3%) [24, 51, 80, 126, 131, 133, 141, 156]; environmental management (*n* = 6; 12.2%) [25, 87, 95, 104, 142, 145]; health system preparation (*n* = 4; 8.2%) [27, 57, 102, 121]; and public education (*n* = 3; 6.1%) [86, 113, 118]. A brief overview table of identified adaptation strategies is provided (Additional file 2), with details accessible on the project website under “Detailed adaptation strategies”.

#### Monitoring and surveillance

Most studies reviewed highlight that monitoring and surveillance are the most critical means of preventing and controlling the spread of WNV under climate change scenarios. Specifically, surveillance should concentrate on high-risk populations, vector populations, wildlife and domestic animals, migrating birds, and neglected areas. As Skaff et al. noted, identifying consistencies between highly susceptible communities and local climates approaching critical thermal thresholds can enhance infectious disease prevention efficacy amidst climate change [75]. Additionally, Semenza et al. recommended fortifying epidemiological monitoring for neuroinvasive diseases potentially indicative of WNV to expand healthcare provider awareness of clinical manifestations and strengthen diagnostic testing capabilities [23]. They also advised augmenting blood donation screening and transportation safeguards while at the same time accounting for climate change in formulating robust WNV contamination prevention protocols [23]. Moreover, numerous studies have suggested that a more granular analysis of meteorological and entomological factors could improve comprehension of intricate WNV transmission dynamics [31, 56, 65, 101, 106, 151, 152]. Concurrently, research and control programs must localize to maximize relevance for regional climate change impacts [101]. Furthermore, public health agencies and vector control teams should amplify efforts to continuously track distributions to minimize human infection risks [151, 152]. Meanwhile, WNV surveillance systems should be strengthened with host monitoring and regular risk assessment, especially for rural livestock, long-distance migratory birds, and wildlife with high mobility [117, 123]. Domestic livestock, particularly horses in high-risk areas, should be vaccinated to enhance their immunity and prevent mortality and morbidity [85]. Routine surveillance should also be conducted in neglected areas (e.g., areas thought not to be transmitted zones and poor areas) [22]. Based on the results of data analysis from surveillance and monitoring, preventive and control strategies need to be adjusted accordingly to cope with changing infectious diseases.

#### Predictive models

Beyond intensifying surveillance, advancing predictive models and early warning systems remain vital for honing outbreak preparedness and rapid response. Sophisticated predictive tools enabling localized risk projections and efficient resource allocation can dramatically amplify intervention impact [38]. Ideally, such systems would synthesize meteorological, biological, genetic, ecological, entomological, and epidemiological data for accurate emergence prediction across scales [49, 70, 74, 81, 84, 103, 105, 111]. Developing predictive models by linking laboratory-observed environmental transmission patterns to actual transmission patterns is crucial to accurately predicting the impact of climate change on WNV and other vector-borne pathogens [49]. Most importantly, next-generation frameworks must address substantial knowledge gaps around viral evolution, vector-host mutations, species migration and adaptation capacity, infection-recovery dynamics, and anthropogenic environmental change impacts on virus shifting dynamics [70, 103, 111]. Advancing models encompassing this intrinsic biocomplexity and policy-environment feedback remains essential to preempt unprecedented post-climate change outbreaks through context-specific preparation and response. International alliances should prioritize pioneering these innovations in prediction science alongside flexible surveillance strengthening for integrated epidemic resilience. Beyond informing ongoing emergence, these efforts will uncover complex ecological interconnectivity in the face of convergence across climate and global changes.

#### Cross-disciplinary/border cooperation

As climate change accelerates, advanced WNV prevention and control requires integrating “One Health” approaches across human, veterinary, wildlife, and environmental health sectors. Multidisciplinary collaboration enables the holistic elucidation of shifting transmission dynamics for accurate risk prediction, alert activation, and adaptive response [126, 133]. Specifically, increased data sharing between public health, vector control, and meteorological agencies, coupled with artificial intelligence integration, can exponentially improve monitoring sensitivity, early warning trigger development, and outbreak interception agility [24, 80]. Additionally, transregional information exchange and coordination remain imperative for refining control strategies and resource allocation amidst climate and global change [156]. The 2018 European WNV emergency exemplified the superiority of integrated “One Health” surveillance, ensuring targeted data-driven countermeasures, bridging counties halted uncontrolled cross-border transmission [141]. Given the existential threat of vector-borne diseases necessitates all governmental and international institutions prioritizing and operationalizing such interdisciplinary preparedness and response architectures. This obligation will grow increasingly urgent as environments continue transforming unprecedentedly.

#### Other adaptive strategies

Of all the studies reviewed, there are fewer strategies related to public education, environmental management, and health system preparedness. However, adapting to the growing threat of WNV under climate change will require multifaceted strategies across environmental management, public awareness-raising, and health system preparedness. Effective environmental management to suppress vector populations, including the elimination of mosquito breeding grounds and the establishment of secondary conserved populations for possible vaccination, forms a crucial first line of defense against WNV [25, 104, 142]. However, this must be coupled with sustained public education campaigns to promote protective behaviors among individuals and vigilant surveillance efforts to enable early response [86, 113, 118]. Finally, health systems must enhance their capacity for detecting WNV outbreaks in vectors and hosts, allowing timely intervention measures, as well as boosting clinical diagnosis and treatment capacity [102, 121].

#### Future work

While this review concentrated on the climatic aspects of WNV transmission, it sets the stage for subsequent in-depth analyses of adaptation strategies within the public health domain. Future studies could adopt a One Health approach or leverage the UNEP framework to explore diverse responses to WNV, thereby enriching the dialogue between climate science and public health policy.

## Conclusions

Climate change may affect the transmission and distribution of WNV, with the extent of the impact depending on local and regional conditions. Surveillance and monitoring stand out as the most recommended adaptation tactics to address the spread of WNV under climate change scenarios. However, far fewer studies have explicitly focused on adaptation strategies than have investigated the impacts of climate change. Further research on the impacts of climate change and adaptation strategies for vector-borne diseases, as well as more comprehensive evidence synthesis, are needed to inform effective policy responses tailored to local contexts.

Our findings highlight the significant role of climate factors in the transmission dynamics of WNV. However, acknowledging the limitations of our focus, we propose future research to extensively explore adaptation strategies that address these climatic challenges. Such efforts would provide comprehensive insights that are crucial for the development of robust public health policies.

### Supplementary Information

Supplementary Material 1.

Supplementary Material 2.

## Data Availability

All data generated or analysed during this study are included in this published article and its supplementary information files.

## Abbreviations

WNV: West Nile virus
IPCC: Intergovernmental Panel on Climate Change
UNEP: United Nations Environmental Program

## References

[1] Kramer LD, Ciota AT, Kilpatrick AM (2019). Introduction, spread, and establishment of West Nile virus in the Americas. J Med Entomol.

[2] Bernkopf H, Levine S, Nerson R (1953). Isolation of West Nile virus in Israel. Infect Dis.

[3] Murgue B, Zeller H, Deubel V (2002). The ecology and epidemiology of West Nile virus in Africa, Europe and Asia. Curr Top Microbiol Immunol.

[4] Johnson N, de FernándezMarco M, Giovannini A (2018). Emerging mosquito-borne threats and the response from European and Eastern Mediterranean countries. Int J Environ Res Public Health.

[5] Nash D, Mostashari F, Fine A, Miller J, O’leary D, Murray K (2001). The outbreak of West Nile virus infection in the New York City area in 1999. N Engl J Med..

[6] Petersen LR, Hayes EB (2008). West Nile virus in the Americas. Med Clin North Am.

[7] Lindsey NP, Staples JE, Lehman JA, Fischer M (2010). Surveillance for human West Nile virus disease-United States, 1999–2008. MMWR Surveill Summ.

[8] Haussig JM, Young JJ, Gossner CM, Mezei E, Bella A, Sirbu A (2018). Early start of the West Nile fever transmission season 2018 in Europe. Euro Surveill.

[9] Camp JV, Nowotny N (2020). The knowns and unknowns of West Nile virus in Europe: what did we learn from the 2018 outbreak?. Expert Rev Anti-Infect Ther.

[10] Pietsch C, Michalski D, Münch J, Petros S, Bergs S, Trawinski H (2020). Autochthonous West Nile virus infection outbreak in humans, Leipzig, Germany, August to September 2020. Euro Surveill.

[11] Vlaskamp DRM, Thijsen SFT, Reimerink J, Hilkens P, Bouvy WH, Bantjes SE (2020). First autochthonous human West Nile virus infections in the Netherlands, July to August 2020. Euro Surveill.

[12] Kramer LD, Styer LM, Ebel GD (2008). A global perspective on the epidemiology of West Nile virus. Annu Rev Entomol.

[13] Kilpatrick AM, Ladeau SL, Marra PP (2007). Ecology of West Nile virus transmission and its impact on birds in the western hemisphere. Auk.

[14] Gómez A, Kilpatrick AM, Kramer LD, Dupuis AP, Maffei JG, Goetz SJ (2008). Land use and West Nile virus seroprevalence in wild mammals. Emerg Infect Dis.

[15] David S, Abraham AM (2016). Epidemiological and clinical aspects on West Nile virus, a globally emerging pathogen. Infect Dis.

[16] Centers for Disease Control. West Nile virus - statistics & maps in 2018. https://www.cdc.gov/westnile/statsmaps/index.html. Accessed 13 June 2023.

[17] Ciota AT (2017). West Nile virus and its vectors. Curr Opin Insect Sci.

[18] Kilpatrick AM (2011). Globalization, land use, and the invasion of West Nile virus. Science.

[19] Jia Y, Moudy RM, Dupuis AP, Ngo KA, Maffei JG, Jerzak GV (2007). Characterization of a small plaque variant of West Nile virus isolated in New York in 2000. Virology.

[20] Kunkel KE, Novak RJ, Lampman RL, Gu W (2006). Modeling the impact of variable climatic factors on the crossover of *Culex restauns* and *Culex pipiens* (Diptera: Culicidae), vectors of West Nile virus in Illinois. Am J Trop Med Hyg.

[21] Watts N, Amann M, Arnell N, Ayeb-Karlsson S, Beagley J, Belesova K (2021). The 2020 report of The Lancet Countdown on health and climate change: responding to converging crises. Lancet.

[22] Harrigan RJ, Thomassen HA, Buermann W, Smith T (2014). A continental risk assessment of West Nile virus under climate change. Global Change Biol.

[23] Semenza JC, Tran A, Espinosa L, Sudre B, Domanovic D, Paz S (2016). Climate change projections of West Nile virus infections in Europe: implications for blood safety practices. Environ Health.

[24] Lorenz C, de Azevedo TS, Chiaravalloti-Neto F (2022). Impact of climate change on West Nile virus distribution in South America. T Roy Soc Trop Med H.

[25] Walker BL, Naugle DE, Doherty KE, Cornish TE (2007). West Nile virus and greater sage-grouse: estimating infection rate in a wild bird population. Avian Dis.

[26] Ziegler U, Lühken R, Keller M, Cadar D, van der Grinten E, Michel F (2019). West Nile virus epizootic in Germany, 2018. Antivir Res.

[27] Keyel AC (2023). Patterns of West Nile virus in the Northeastern United States using negative binomial and mechanistic trait-based models. GeoHealth.

[28] Keyel AC, Elison Timm O, Backenson PB, Prussing C, Quinones S, McDonough KA (2019). Seasonal temperatures and hydrological conditions improve the prediction of West Nile virus infection rates in Culex mosquitoes and human case counts in New York and Connecticut. Plos One.

[29] Chersich MF, Wright CY (2019). Climate change adaptation in South Africa: a case study on the role of the health sector. Global Health.

[30] Bardosh KL, Ryan S, Ebi K, Welburn S, Singer B (2017). Addressing vulnerability, building resilience: community-based adaptation to vector-borne diseases in the context of global change. Infect Dis Pover.

[31] Cox R, Sanchez J, Revie CW (2013). Multi-criteria decision analysis tools for prioritising emerging or re-emerging infectious diseases associated with climate change in Canada. Plos One.

[32] Hongoh V, Michel P, Gosselin P, Samoura K, Ravel A, Campagna C (2016). Multi-stakeholder decision aid for improved prioritization of the public health impact of climate sensitive infectious diseases. Int J Environ Heal R.

[33] Pachauri RK, Reisinger A (2007). Climate Change 2007: synthesis Report. Contribution of working groups I, II and III to the fourth assessment report of the intergovernmental panel on climate change.

[34] Pachauri RK, Allen MR, Barros VR, Broome J, Cramer W, Christ R, Climate change, (2014). Synthesis Report, Contribution of Working Groups I, II and III to the Fifth Assessment Report of the Intergovernmental Panel on Climate Change. IPCC..

[35] Watts N, Adger WN, Agnolucci P (2015). Health and climate change: policy responses to protect public health. Lancet.

[36] Eder M, Cortes F, de SiqueiraFilhaTeixeira N, de Franca Araújo GV, Degroote S, Braga C (2018). Scoping review on vector-borne diseases in urban areas: transmission dynamics, vectorial capacity and co-infection. Infect Dis Poverty.

[37] Orr M, Inoue Y, Seymour R, Dingle G (2022). Impacts of climate change on organized sport: a scoping review. WIREs Clim Change.

[38] Kulkarni MA, Duguay C, Ost K (2022). Charting the evidence for climate change impacts on the global spread of malaria and dengue and adaptive responses: a scoping review of reviews. Global Health.

[39] Schultz A, Goertzen L, Rothney J, Wener P, Enns J, Halas G (2018). A scoping approach to systematically review published reviews: adaptations and recommendations. Res Synth Methods.

[40] Tricco AC, Lillie E, Zarin W, O'Brien KK, Colquhoun H, Levac D (2018). PRISMA extension for scoping reviews (PRISMA-ScR): checklist and explanation. Ann Intern Med.

[41] Sweileh WM (2020). Bibliometric analysis of peer-reviewed literature on climate change and human health with an emphasis on infectious diseases. Glob Health.

[42] Masson-Delmotte VP, Zhai P, Pirani SL, Connors C, Péan S, Berger N (2021). IPCC. Summary for Policymakers. Climate change 2021: The Physical Science Basis. Contribution of Working Group I to the Sixth Assessment Report of the Intergovernmental Panel on Climate Change.

[43] IPCC. An IPCC Special Report on the impacts of global warming of 1.5°C above preindustrial levels and related global greenhouse gas emission pathways, in the context of strengthening the global response to the threat of climate change, sustainable development, and efforts to eradicate poverty. https://pure.iiasa.ac.at/id/eprint/15716/1/SR15_TS_High_Res.pdf. Accessed 29 Jan 2019.

[44] Feenstra JF, Burton I, Smith JB, Tol RS (1998). Handbook on Methods for Climate Change Impact Assessment and Adaptation Strategies.

[45] Isaiah PM, Sólveig Palmeirim M, Steinmann P (2023). Epidemiology of pediatric schistosomiasis in hard-to-reach areas and populations: a scoping review. Infect Dis Poverty.

[46] Wang H, Guo T, Wang Z, Xiao J, Gao L, Gao X (2023). PreCowKetosis: A Shiny web application for predicting the risk of ketosis in dairy cows using prenatal indicators. Comput Electron Agr.

[47] Soverow JE, Wellenius GA, Fisman DN, Mittleman MA (2009). Infectious disease in a warming world: how weather influenced West Nile virus in the United States (2001–2005). Environ Health Persp.

[48] Wang G, Minnis RB, Belant JL, Wax CL (2010). Dry weather induces outbreaks of human West Nile virus infections. BMC Infect Dis.

[49] Kilpatrick AM, Meola MA, Moudy RM, Kramer LD (2008). Temperature, viral genetics, and the transmission of West Nile virus by Culex pipiens mosquitoes. Plos Pathog.

[50] LaDeau SL, Calder CA, Doran PJ, Marra PP (2011). West Nile virus impacts in American crow populations are associated with human land use and climate. Ecol Res..

[51] Liu H, Weng Q (2012). Environmental factors and risk areas of West Nile Virus in southern California, 2007–2009. Environ Model Assess.

[52] Walsh MG (2012). The role of hydrogeography and climate in the landscape epidemiology of West Nile virus in New York State from 2000 to 2010. Plos One.

[53] Landesman WJ, Allan BF, Langerhans RB, Knight TM, Chase JM (2007). Inter-annual associations between precipitation and human incidence of West Nile virus in the United States. Vector-Borne Zoonot.

[54] Wimberly MC, Hildreth MB, Boyte SP, Lindquist E, Kightlinger L (2008). Ecological niche of the 2003 West Nile virus epidemic in the northern Great Plains of the United States. Plos One.

[55] Deichmeister JM, Telang A (2011). Abundance of West Nile virus mosquito vectors in relation to climate and landscape variables. J Vector Ecol.

[56] DeGroote JP, Sugumaran R, Brend SM, Tucker BJ, Bartholomay LC (2008). Landscape, demographic, entomological, and climatic associations with human disease incidence of West Nile virus in the state of Iowa, USA. Int J Health Geogr.

[57] Shaman J, Harding K, Campbell SR (2011). Meteorological and hydrological influences on the spatial and temporal prevalence of West Nile virus in *Culex* mosquitoes, Suffolk County. New York J Med Entomol.

[58] Winters AM, Eisen RJ, Lozano-Fuentes S, Moore CG, Pape WJ, Eisen L (2008). Predictive spatial models for risk of West Nile virus exposure in eastern and western Colorado. Am J Trop Med Hyg.

[59] Chuang TW, Wimberly MC (2012). Remote sensing of climatic anomalies and West Nile virus incidence in the northern Great Plains of the United States. Plos One.

[60] Hartley DM, Barker CM, Le Menach A, Niu T, Gaff HD, Reisen WK (2012). Effects of temperature on emergence and seasonality of West Nile virus in California. Am J Trop Med Hyg.

[61] Ruiz MO, Chaves LF, Hamer GL, Sun T, Brown WM, Walker ED (2010). Local impact of temperature and precipitation on West Nile virus infection in *Culex* species mosquitoes in northeast Illinois, USA. Parasites Vector.

[62] Shaman J, Day JF, Komar N (2010). Hydrologic conditions describe West Nile virus risk in Colorado. Int J Environ Res Public He.

[63] Epp TY, Waldner C, Berke O (2011). Predictive risk mapping of West Nile virus (WNV) infection in Saskatchewan horses. Can J Vet Res.

[64] Roth D, Henry B, Mak S, Fraser M, Taylor M, Li M (2010). West Nile virus range expansion into British Columbia. Emerg Infect Dis.

[65] Platonov AE, Tolpin VA, Gridneva KA, Titkov AV, Platonova OV, Kolyasnikova NM (2014). The incidence of West Nile disease in Russia in relation to climatic and environmental factors. Int J Environ Heal R.

[66] Paz S, Albersheim I (2008). Influence of warming tendency on *Culex pipiens* population abundance and on the probability of West Nile fever outbreaks (Israeli case study:2001–2005). EcoHealth.

[67] Uejio CK, Kemp A, Comrie AC (2012). Climatic controls on West Nile virus and Sindbis virus transmission and outbreaks in South Africa. Vector-Borne Zoonot.

[68] Chaves LF, Hamer GL, Walker ED, Brown WM, Ruiz MO, Kitron UD (2011). Climatic variability and landscape heterogeneity impact urban mosquito diversity and vector abundance and infection. Ecosphere.

[69] Hongoh V, Berrang-Ford L, Scott ME, Lindsay LR (2012). Expanding geographical distribution of the mosquito, Culex pipiens, in Canada under climate change. Appl Geogr.

[70] Gale P, Brouwer A, Ramnial V, Kelly L, Kosmider R, Fooks AR (2010). Assessing the impact of climate change on vector-borne viruses in the EU through the elicitation of expert opinion. Epidemiol Infect.

[71] Paz S, Malkinson D, Green MS, Tsioni G, Papa A, Danis K (2013). Permissive summer temperatures of the 2010 European West Nile fever upsurge. Plos One.

[72] Johnson BJ, Sukhdeo MVK (2013). Drought-induced amplification of local and regional West Nile virus infection rates in New Jersey. J Med Entomol.

[73] Ukawuba I, Shaman J (2018). Association of spring-summer hydrology and meteorology with human West Nile virus infection in West Texas, USA, 2002–2016. Parasites Vector.

[74] Little E, Campbell SR, Shaman J (2016). Development and validation of a climate-based ensemble prediction model for West Nile virus infection rates in Culex mosquitoes, Suffolk County New York. Parasites Vector.

[75] Skaff NK, Cheng Q, Clemesha RES, Collender PA, Gershunov A, Head JR (1932). Thermal thresholds heighten sensitivity of West Nile virus transmission to changing temperatures in coastal California. P Roy Soc B-Biol Sci.

[76] Shocket MS, Verwillow AB, Numazu MG, Slamani H, Cohen JM, El Moustaid F (2020). Transmission of West Nile and five other temperate mosquito-borne viruses peaks at temperatures between 23 C and 26 C. Elife.

[77] Crowder DW, Dykstra EA, Brauner JM, Duffy A, Reed C, Martin E (2013). West Nile virus prevalence across landscapes is mediated by local effects of agriculture on vector and host communities. Plos One.

[78] Smith KH, Tyre AJ, Hamik J, Hayes MJ, Zhou Y, Dai L (2020). Using climate to explain and predict West Nile Virus risk in Nebraska. GeoHealth..

[79] Tokarz RE, Smith RC (2020). Crossover dynamics of *Culex* (Diptera: Culicidae) vector populations determine WNV transmission intensity. J Med Entomol.

[80] Lockaby G, Noori N, Morse W, Zipperer W, Kalin L, Governo R (2016). Climatic, ecological, and socioeconomic factors associated with West Nile virus incidence in Atlanta, Georgia, USA. J Vector Ecol.

[81] Uelmen JA, Brokopp C, Patz J (2020). A 15 year evaluation of West Nile Virus in Wisconsin: effects on wildlife and human health. Int J Environ Heal R.

[82] Hahn MB, Monaghan AJ, Hayden MH, Eisen RJ, Delorey MJ, Lindsey NP (2015). Meteorological conditions associated with increased incidence of West Nile virus disease in the United States, 2004–2012. Am J Trop Med Hyg.

[83] Wimberly MC, Lamsal A, Giacomo P, Chuang TW (2014). Regional variation of climatic influences on West Nile virus outbreaks in the United States. Am J Trop Med Hyg.

[84] Fay RL, Ngo KA, Kuo L, Willsey GG, Kramer LD, Ciota AT (2021). Experimental evolution of West Nile virus at higher temperatures facilitates broad adaptation and increased genetic diversity. Viruses.

[85] Humphreys JM, Pelzel-McCluskey AM, Cohnstaedt LW, McGregor BL, Hanley KA, Hudson AR (2021). Integrating spatiotemporal epidemiology, eco-phylogenetics, and distributional ecology to assess West Nile disease risk in horses. Viruses.

[86] Hernandez E, Torres R, Joyce AL (2019). Environmental and sociological factors associated with the incidence of West Nile virus cases in the Northern San Joaquin Valley of California, 2011–2015. Vector-Borne Zoonot.

[87] Myer MH, Campbell SR, Johnston JM (2017). Spatiotemporal modeling of ecological and sociological predictors of West Nile virus in Suffolk County, NY, mosquitoes. Ecosphere.

[88] Myer MH, Johnston JM (2019). Spatiotemporal Bayesian modeling of West Nile virus: Identifying risk of infection in mosquitoes with local-scale predictors. Sci Total Environ.

[89] Kala AK, Tiwari C, Mikler AR, Atkinson SF (2017). A comparison of least squares regression and geographically weighted regression modeling of West Nile virus risk based on environmental parameters. Peer J.

[90] Day JF, Shaman J (2014). Using hydrologic conditions to forecast the risk of focal and epidemic arboviral transmission in peninsular Florida. J Med Entomol.

[91] Peper ST, Dawson DE, Dacko N, Athanasiou K, Hunter J, Loko F (2018). Predictive modeling for West Nile virus and mosquito surveillance in Lubbock Texas. J Am Mosquito Contr.

[92] Poh KC, Chaves LF, Reyna-Nava M, Roberts CM, Fredregill C, Bueno R (2019). The influence of weather and weather variability on mosquito abundance and infection with West Nile virus in Harris County, Texas, USA. Sci Total Environ..

[93] Shand L, Brown WM, Chaves LF, Goldberg TL, Hamer GL, Haramis L (2016). Predicting West Nile virus infection risk from the synergistic effects of rainfall and temperature. J Med Entomol.

[94] Mori H, Wu J, Ibaraki M, Schwartz FW (2018). Key factors influencing the incidence of West Nile virus in Burleigh County, North Dakota. Int J Environ Res Public He.

[95] Ward MJ, Sorek-Hamer M, Henke JA, Little E, Patel A, Shaman J (2023). A spatially resolved and environmentally informed forecast model of West Nile virus in Coachella Valley, California. GeoHealth.

[96] Gorris ME, Randerson JT, Coffield SR, Treseder KK, Zender CS, Xu C, Manore CA (2023). Assessing the influence of climate on the spatial pattern of West Nile virus incidence in the United States. Environ Health Persp.

[97] Huang X, Athrey GN, Kaufman PE, Fredregill C, Slotman MA (2023). Effective population size of *Culex quinquefasciatus* under insecticide-based vector management and following Hurricane Harvey in Harris County Texas. Front Genet.

[98] Holcomb KM, Mathis S, Staples JE, Fischer M, Barker CM, Beard CB (2023). Evaluation of an open forecasting challenge to assess skill of West Nile virus neuroinvasive disease prediction. Parasite Vector.

[99] Paull SH, Horton DE, Ashfaq M, Rastogi D, Kramer LD, Diffenbaugh NS (1848). Drought and immunity determine the intensity of West Nile virus epidemics and climate change impacts. P Roy Soc B-Biol Sci.

[100] Keyel AC, Raghavendra A, Ciota AT, Elison TO (2021). West Nile virus is predicted to be more geographically widespread in New York State and Connecticut under future climate change. Global Change Biol.

[101] Morin CW, Comrie AC (2013). Regional and seasonal response of a West Nile virus vector to climate change. P Natl A Sci.

[102] Filippelli GM, Freeman JL, Gibson J, Jay S, Moreno-Madriñán MJ, Ogashawara I (2020). Climate change impacts on human health at an actionable scale: a state-level assessment of Indiana, USA. Clim Change.

[103] Brown HE, Young A, Lega J, Andreadis TG, Schurich J, Comrie A (2015). Projection of climate change influences on US West Nile virus vectors. Earth Interact.

[104] Bakker VJ, Sillett TS, Boyce WM, Doak DF, Vickers TW, Reisen WK (2020). Translocation with targeted vaccination is the most effective strategy to protect an island endemic bird threatened by West Nile virus. Divers Distrib.

[105] Chen CC, Epp T, Jenkins E, Waldner C, Curry PS, Soos C (2013). Modeling monthly variation of
* Culex tarsalis
* (Diptera:Culicidae) abundance and West Nile Virus infection rate in the Canadian Prairies. Int J Environ Heal R.

[106] Mallya S, Sander B, Roy-Gagnon MH, Taljaard M, Jolly A, Kulkarn MA (2018). Factors associated with human West Nile virus infection in Ontario: a generalized linear mixed modelling approach. BMC Infect Dis.

[107] Temple SD, Manore CA, Kaufeld KA (2022). Bayesian time-varying occupancy model for West Nile virus in Ontario Canada. Stoch Env Res Risk A.

[108] Talbot B, Kulkarni MA, Rioux-Rousseau M, Siebels K, Kotchi SO, Ogden NH (2023). Ecological niche and positive clusters of two West Nile virus vector in Ontario Canada. EcoHealth.

[109] Albrecht L, Kaufeld KA (2023). Investigating the impact of environmental factors on West Nile virus human case prediction in Ontario Canada. Front Public Health.

[110] Baril C, Pilling BG, Mikkelsen MJ, Sparrow JM, Duncan CAM, Koloski CW (2023). The influence of weather on the population dynamics of common mosquito vector species in the Canadian Prairies. Parasite Vector.

[111] Chen CC, Jenkins E, Epp T, Waldner C, Curry PS, Soos C (2013). Climate change and West Nile virus in a highly endemic region of North America. Int J Environ Heal R.

[112] Otten A, Fazil A, Chemeris A, Breadner P, Ng V (2020). Prioritization of vector-borne diseases in Canada under current climate and projected climate change. Microbial Risk Anal.

[113] Hongoh V, Campagna C, Panic M, Samuel O, Gosselin P, Waaub JP (2016). Assessing interventions to manage West Nile virus using multi-criteria decision analysis with risk scenarios. Plos One.

[114] Tam BY, Tsuji LJS (2016). West Nile virus in American crows (*Corvus brachyrhynchos*) in Canada: projecting the influence of climate change. GeoJournal.

[115] Tam BY, Martin I, Tsuji LJS (2014). Geospatial analysis between the environment and past incidences of West Nile virus in bird specimens in Ontario Canada. GeoJournal.

[116] Rakotoarinia MR, Seidou O, Lapen DR, Leighton PA, Ogden NH, Ludwig A (2023). Future land-use change predictions using Dyna-Clue to support mosquito-borne disease risk assessment. Environ Monit Assess.

[117] Di Pol G, Crotta M, Taylor RA (2022). Modelling the temperature suitability for the risk of West Nile Virus establishment in European *Culex pipiens* populations. Transbound Emerg Dis..

[118] Coroian M, Petrić M, Pistol A, Sirbu A, Domșa C, Mihalca AD (2020). Human West Nile Meningo-Encephalitis in a highly endemic country: a complex epidemiological analysis on biotic and abiotic risk factors. Int J Environ Heal R.

[119] Stilianakis NI, Syrris V, Petroliagkis T, Pärt P, Gewehr S, Kalaitzopoulou S (2016). Identification of climatic factors affecting the epidemiology of human West Nile virus infections in northern Greece. Plos One.

[120] Vogels CB, Hartemink N, Koenraadt CJM (2017). Modelling West Nile virus transmission risk in Europe: effect of temperature and mosquito biotypes on the basic reproduction number. Sci Rep.

[121] Fros JJ, Geertsema C, Vogels CB, Roosjen PP, Failloux AB, Vlak JM (2015). West Nile virus: high transmission rate in north-western European mosquitoes indicates its epidemic potential and warrants increased surveillance. Plos Neglect Trop D.

[122] Tran A, Sudre B, Paz S, Rossi M, Desbrosse A, Chevalier V (2014). Environmental predictors of West Nile fever risk in Europe. Int J Health Geogr.

[123] Radojicic S, Zivulj A, Petrovic T, Nisavic J, Milicevic V, Sipetic-Grujicic S (2021). Spatiotemporal analysis of West Nile virus epidemic in South Banat District, Serbia, 2017–2019. Animals.

[124] Platonov AE, Fedorova MV, Karan LS, Shopenskaya TA, Platonova OV, Zhuravlev VI (2008). Epidemiology of West Nile infection in Volgograd, Russia, in relation to climate change and mosquito (Diptera: Culicidae) bionomics. Parasitol Res.

[125] Moirano G, Gasparrini A, Acquaotta F, Fratianni S, Merletti F, Maule M (2018). West Nile virus infection in Northern Italy: Case-crossover study on the short-term effect of climatic parameters. Environ Res.

[126] Marcantonio M, Rizzoli A, Metz M, Rosà R, Marini G, Chadwick E (2015). Identifying the environmental conditions favouring West Nile virus outbreaks in Europe. Plos One.

[127] Mihailović DT, Petrić D, Petrović T, Hrnjaković-Cvjetković I, Djurdjevic V, Nikolić-Đorić E (2020). Assessment of climate change impact on the malaria vector *Anopheles hyrcanus,* West Nile disease, and incidence of melanoma in the Vojvodina Province (Serbia) using data from a regional climate model. Plos One.

[128] Trájer AJ, Bede-Fazekas Á, Bobvos J, Páldy A (2014). Seasonality and geographical occurrence of West Nile fever and distribution of Asian tiger mosquito. Q J Hung Meteorol Se.

[129] Townroe S, Callaghan A (2014). British container breeding mosquitoes: the impact of urbanisation and climate change on community composition and phenology. Plos One.

[130] Paz S (2012). West Nile Virus Eruptions in Summer 2010–What Is the Possible Linkage with Climate Change?.

[131] Mavrakis A, Papavasileiou C, Alexakis D, Papakitsos EC, Salvati L (2021). Meteorological patterns and the evolution of West Nile virus in an environmentally stressed Mediterranean area. Environ Monit Assess.

[132] Vlasova NV, Masyagutova LM, Abdrakhmanova ER, Rafikova LA, Chudnovets GM (2022). A conceptual scheme of a predictive-analytical model for describing incidence of west nile fever based on weather and climate estimation (exemplified by the Volgograd region). Health Risk Anal.

[133] Farooq Z, Rocklöv J, Wallin J, Abiri N, Sewe MO, Sjödin H (2022). Artificial intelligence to predict West Nile virus outbreaks with ecoclimatic drivers. Lancet Reg Health Eu.

[134] Marini G, Pugliese A, Wint W, Alexander NS, Rizzoli A, Rosà R (2022). Modelling the West Nile virus force of infection in the European human population. One Health.

[135] Vukmir NR, Bojanić J, Mijović B, Roganović T, Aćimović J (2019). Did intensive floods influence higher incidence rate of the West Nile virus in the population exposed to flooding in the republic of Srpska in 2014. Arch Vet Med.

[136] Krol L, Blom R, Dellar M, van der Beek JG, Stroo ACJ, van Bodegom PM (2023). Interactive effects of climate, land use and soil type on *Culex pipiens/torrentium* abundance. One Health.

[137] Magallanes S, Llorente F, Ruiz-López MJ,  de la PuenteMartinez - J, Soriguer R, Calderon J (2023). Long-term serological surveillance for West Nile and Usutu virus in horses in south-West Spain. One Health.

[138] Niczyporuk JS, Kozdrun W, Czujkowska A, Blanchard Y, Helle M, Dheilly NM (2023). West Nile virus lineage 2 in free-living *Corvus cornix* birds in Poland. Trop Med Infect Dis.

[139] Angelou A, Gewehr S, Mourelatos S, Kioutsioukis I (2023). Early warning impact of temperature and rainfall anomalies onto West Nile virus human cases. Environ Sci Proc.

[140] Watts MJ, iMonteys VS, Mortyn PG, Kotsila P (2021). The rise of West Nile virus in Southern and Southeastern Europe: A spatial–temporal analysis investigating the combined effects of climate, land use and economic changes. One Health..

[141] Lourenço J, Barros SC, Zé-Zé L, Damineli DSC, Giovanetti M, Osório HC (2022). West Nile virus transmission potential in Portugal. Comms Biol.

[142] Ewing DA, Purse BV, Cobbold CA, White SM (2021). A novel approach for predicting risk of vector-borne disease establishment in marginal temperate environments under climate change: West Nile virus in the UK. J R Soc Interface.

[143] Trájer AJ (2017). Meteorological conditions associated with West Nile fever incidences in mediterranean and continental climates in Europe. Idojaras.

[144] Tippelt L, Walther D, Kampen H (2017). The thermophilic mosquito species *Uranotaenia unguiculata* Edwards, 1913 (Diptera: Culicidae) moves north in Germany. Parasitol Res.

[145] Farooq Z, Sjödin H, Semenza JC, Tozan Y, Sewe MO, Wallin J, Rocklöv J (2023). European projections of West Nile virus transmission under climate change scenarios. One Health.

[146] Aharonson-Raz K, Lichter-Peled A, Tal S, Gelman B, Cohen D, Klement E (2014). Spatial and temporal distribution of west Nile virus in horses in Israel (1997–2013)-From endemic to epidemics. Plos One.

[147] Ahmadnejad F, Otarod V, Fathnia A, Ahmadabadi A, Fallah MH, Zavareh A (2016). Impact of climate and environmental factors on West Nile virus circulation in Iran. J Arthropod-Borne Di.

[148] Salama M, Amitai Z, Lustig Y, Mor Z, Weiberger M, Chowers M (2018). Outbreak of West Nile virus disease in Israel (2015): A retrospective analysis of notified cases. Travel Med Infect Di.

[149] Calistri P, Ippoliti C, Candeloro L, Benjelloun A, El Harrak M, Bouchra B (2013). Analysis of climatic and environmental variables associated with the occurrence of West Nile virus in Morocco. Prev Vet Med.

[150] Velu RM, Kwenda G, Bosomprah S, Chisola MN, Simunyandi M, Chisenga CC (2023). Ecological niche modeling of *Aedes* and *Culex* mosquitoes: a risk map for Chikungunya and West Nile viruses in Zambia. Viruses.

[151] Outammassine A, Zouhair S, Loqman S (2022). Rift Valley fever and West Nile virus vectors in Morocco: Current situation and future anticipated scenarios. Transbound Emerg Dis.

[152] Figueroa DP, Scott S, González CR, Bizama G, Flores Mara R, Bustamante R (2020). Estimating the climate change consequences on the potential distribution of Culex pipiens l. 1758, to assess the risk of West Nile virus establishment in Chile. Gatana.

[153] Huang B, Prow NA, van den Hurk AF, Allcock RJ, Moore PR, Doggett SL (2016). Archival isolates confirm a single topotype of West Nile virus in Australia. Plos Neglected Trop D.

[154] Anyamba A, Small JL, Britch SC, Tucker CJ, Pak EW, Reynolds CA (2014). Recent weather extremes and impacts on agricultural production and vector-borne disease outbreak patterns. Plos One.

[155] Samy AM, Elaagip AH, Kenawy MA, Ayres CF, Peterson AT, Soliman DE (2016). Climate change influences on the global potential distribution of the mosquito *Culex quinquefasciatus*, vector of West Nile virus and lymphatic filariasis. Plos One.

[156] Negev M, Paz S, Clermont A, Pri-Or NG, Shalom U, Yeger T (2015). Impacts of climate change on vector borne diseases in the Mediterranean Basin—implications for preparedness and adaptation policy. Int J Environ Heal R.

[157] CDC. Historic Data for WNV, 1999–2022. https://www.cdc.gov/westnile/statsmaps/historic-data.html. Accessed 11 Oct 2023.

[158] Soto RA, Hughes ML, Staples JE, Lindsey NP (2022). West Nile virus and other domestic nationally notifiable arboviral diseases-United States, 2020. MMWR-Morbid Mortal W.

[159] Zheng H, Drebot MA, Coulthart MB (2014). West Nile virus in Canada: ever-changing, but here to stay Canada. Commun Dis Rep.

[160] Public Health Agency of Canada. West Nile virus and other mosquito-borne disease national surveillance report. https://www.canada.ca/en/public-health/services/diseases/west-nile-virus/surveillance-west-nile-virus.html. Accessed 11 March 2024.

[161] Young JJ, Haussig JM, Aberle SW, Pervanidou D, Riccardo F, Sekulić N (2021). Epidemiology of human West Nile virus infections in the European Union and European Union enlargement countries, 2010 to 2018. Eurosurveillance.

[162] CDC. FAQ: general questions about West Nile virus. 2015. http://www.cdc.gov/westnile/faq/genQuestions.html. Accessed 13 Jun 2023.

[163] Komar N (2003). West Nile virus: epidemiology and ecology in North America. Adv Virus Res.

[164] George TL, Harrigan RJ, LaManna JA, DeSante DF, Saracco JF, Smith TB (2015). Persistent impacts of West Nile virus on North American bird populations. P Natl Acad Sci USA.

[165] Hamer GL, Walker ED, Brawn JD, Loss SR, Ruiz MO, Goldberg TL (2008). Rapid amplification of West Nile virus: the role of hatch-year birds. Vector-Borne Zoonotic Dis.

[166] Reisen WK (1995). Effect of temperature on *Culex tarsalis* (Diptera: Culicidae) from the Coachella and San Joaquin valleys of California. J Med Entomol.

[167] Cotton PA (2003). Avian migration phenology and global climate change. P Natl Acad Sci USA.

[168] Caillouët KA, Riggan AE, Bulluck LP, Carlson JC, Sabo RT (2013). Nesting bird “host funnel” increases mosquito-bird contact rate. J Med Entomol.

[169] Shaman J, Day JF, Stieglitz M (2005). Drought induced amplification and epidemic transmission of West Nile virus in southern Florida. J Med Entomol.

[170] Paz S (2015). Climate change impacts on West Nile virus transmission in a global context. Philos Trans R Soc, B.

