# Supplementary material for: Impact of climate change on the global circulation of West Nile virus and adaptation responses: a scoping review
Source: Infect Dis Poverty. 2024 May 24;13:38. doi: 10.1186/s40249-024-01207-2 (PMC11127377; doi:10.1186/s40249-024-01207-2)
Supplement: Supplementary file 1 — Supplementary Material 1. [file 40249_2024_1207_MOESM1_ESM.docx]

**Additional file 1 Search strategy by database**

**1. Key concepts**

| Climate change | West Nile Virus |
| --- | --- |

**2. Associated keywords:**

| **Climate change** | **West Nile virus** |
| --- | --- |
| Climate change | West Nile virus |
| Changing climate | West Nile fever |
| Global warming | WNV |
| Climate variability | West Nile disease |
| Climate fluctuation | West Nile virus infection |
| Rising temperature | WNV infection |
| Temperature change | Encephalitis, West Nile fever |
| Precipitation change | West Nile fever encephalitis |
| Extreme weather | West Nile fever meningitis |
| Extreme event | West Nile fever meningoencephalitis |
| Extreme temperature | WNV transmiss* |
| Heat wave | WNV vector |
| Drought | WNV host |
| Flood | WNV circulation |
| Sea level rise |  |
| CO_2_ concentration |  |
| Greenhouse effect |  |
| Greenhouse gas |  |
| Anthropogenic forcing |  |
| Natural forcing |  |

And use these words (Transmiss* or Spread* or Circulat* or Distribut* or Disseminat* or Propagat* or Contagion* or Communicat* or Diffus* or Dispers* or Expans* or Outbreak* or Epidemic* or Pandemic* or Prevalen* or Inciden* or Occurren* or Emergen* or Clon* or Persisten* or Infect*) to limit the results.

**3. Databases to search:**

Scientific literature: PubMed, Scopus, Institute for Scientific Information Web of science, EBSCOhost

**Pubmed**

(Climat* [Title/Abstract] or Climate change [Title/Abstract] or Changing climate [Title/Abstract] or Global warming [Title/Abstract] or Climate variability [Title/Abstract] or Climate fluctuation [Title/Abstract] or Rising temperature [Title/Abstract] or Temperature change [Title/Abstract] or Precipitation change [Title/Abstract] or Extreme weather [Title/Abstract] or Extreme event [Title/Abstract] or Extreme temperature [Title/Abstract] or Heat wave [Title/Abstract] or Drought [Title/Abstract] or Flood [Title/Abstract] or Sea level rise [Title/Abstract] or CO2 concentration [Title/Abstract] or Greenhouse effect [Title/Abstract] or Greenhouse gas [Title/Abstract] or Anthropogenic forcing [Title/Abstract] or Natural forcing [Title/Abstract]) and (West Nile Virus [Title/Abstract] or West Nile Fever [Title/Abstract] or WNV [Title/Abstract] or West Nile disease [Title/Abstract] or West Nile virus infection [Title/Abstract] or WNV infection [Title/Abstract] or Encephalitis, West Nile fever [Title/Abstract] or West Nile fever encephalitis [Title/Abstract] or West Nile fever meningitis [Title/Abstract] or West Nile fever meningoencephalitis [Title/Abstract] or WNV transmiss* [Title/Abstract] or WNV vector [Title/Abstract] or WNV host [Title/Abstract] or WNV circulation [Title/Abstract]) and (Transmiss* [Title/Abstract] or Spread* [Title/Abstract] or Circulat* [Title/Abstract] or Distribut* [Title/Abstract] or Disseminat* [Title/Abstract] or Propagat* [Title/Abstract] or Contagion* [Title/Abstract] or Communicat* [Title/Abstract] or Diffus* [Title/Abstract] or Dispers* [Title/Abstract] or Expans* [Title/Abstract] or Outbreak* [Title/Abstract] or Epidemic* [Title/Abstract] or Pandemic* [Title/Abstract] or Prevalen* [Title/Abstract] or Inciden* [Title/Abstract] or Occurren* [Title/Abstract] or Emergen* [Title/Abstract] or Clon* [Title/Abstract] or Persisten* [Title/Abstract] or Infect* [Title/Abstract])

**Scopus**

(TITLE-ABS-KEY ( ("Climat*" or "Climate change" or "Changing climate" or "Global warming" or "Climate variability" or "Climate fluctuation" or "Rising temperature" or "Temperature change" or "Precipitation change" or "Extreme weather" or "Extreme event" or "Extreme temperature" or "Heat wave" or "Drought" or "Flood" or "Sea level rise" or "CO2 concentration" or "Greenhouse effect" or "Greenhouse gas" or "Anthropogenic forcing" or "Natural forcing") ) ) and (TITLE-ABS-KEY ( ( "West Nile Virus" or "West Nile Fever" or "WNV" or "West Nile disease" or "West Nile virus infection" or "WNV infection" or "Encephalitis, West Nile fever" or "West Nile fever encephalitis" or "West Nile fever meningitis" or "West Nile fever meningoencephalitis" or "WNV transmiss*" or "WNV vector" or "WNV host" or "WNV circulation") ) ) and (TITLE-ABS-KEY ( ("Transmiss*" or "Spread*" or "Circulat*" or "Distribut*" or "Disseminat*" or "Propagat*" or "Contagion*" or "Communicat*" or "Diffus*" or "Dispers*" or "Expans*" or "Outbreak*" or "Epidemic*" or "Pandemic*" or "Prevalen*" or "Inciden*" or "Occurren*" or "Emergen*" or "Clon*" or "Persisten*" or "Infect*") ) )

**Web of Science**

TS=(Climat* or Climate change or Changing climate or Global warming or Climate variability or Climate fluctuation or Rising temperature or Temperature change or Precipitation change or Extreme weather or Extreme event or Extreme temperature or Heat wave or Drought or Flood or Sea level rise or CO2 concentration or Greenhouse effect or Greenhouse gas or Anthropogenic forcing or Natural forcing) and TS=(West Nile Virus or West Nile Fever or WNV or West Nile disease or West Nile virus infection or WNV infection or Encephalitis, West Nile fever or West Nile fever encephalitis or West Nile fever meningitis or West Nile fever meningoencephalitis or WNV transmiss* or WNV vector or WNV host or WNV circulation) and TS=(Transmiss* or Spread* or Circulat* or Distribut* or Disseminat* or Propagat* or Contagion* or Communicat* or Diffus* or Dispers* or Expans* or Outbreak* or Epidemic* or Pandemic* or Prevalen* or Inciden* or Occurren* or Emergen* or Clon* or Persisten* or Infect*)

**EBSCOhost**

SU(Climat* or Climate change or Changing climate or Global warming or Climate variability or Climate fluctuation or Rising temperature or Temperature change or Precipitation change or Extreme weather or Extreme event or Extreme temperature or Heat wave or Drought or Flood or Sea level rise or CO2 concentration or Greenhouse effect or Greenhouse gas or Anthropogenic forcing or Natural forcing) and SU(West Nile Virus or West Nile Fever or WNV or West Nile disease or West Nile virus infection or WNV infection or Encephalitis, West Nile fever or West Nile fever encephalitis or West Nile fever meningitis or West Nile fever meningoencephalitis or WNV transmiss* or WNV vector or WNV host or WNV circulation) and SU(Transmiss* or Spread* or Circulat* or Distribut* or Disseminat* or Propagat* or Contagion* or Communicat* or Diffus* or Dispers* or Expans* or Outbreak* or Epidemic* or Pandemic* or Prevalen* or Inciden* or Occurren* or Emergen* or Clon* or Persisten* or Infect*)

**4. Search results**

| Database | Number of documents found |
| --- | --- |
| Pubmed | 396 |
| Web of science | 885 |
| Scopus | 757 |
| EBSCOhost | 130 |
| Total | 2168 |

Records after duplicates removed: 1272
