# Supplementary material for: Impact of climate change on the global circulation of West Nile virus and adaptation responses: a scoping review
Source: Infect Dis Poverty. 2024 May 24;13:38. doi: 10.1186/s40249-024-01207-2 (PMC11127377; doi:10.1186/s40249-024-01207-2)
Supplement: Supplementary file 2 — Supplementary Material 2. [file 40249_2024_1207_MOESM2_ESM.docx]

**Additional file 2** A brief overview table of identified adaptation strategies

| **Surveillance and monitoring** | |
| --- | --- |
| Constant monitoring of population at risk | Enhanced epidemiologic surveillance for neuroinvasive  disease (which may suggest infection with WNV) |
| Maintain focused monitoring of wildlife and domestic animals, especially those kept in rural areas | Measures to survey and monitor vector population  dynamics and climate data |
| Preventive control measures and standard setting | Risk assessment |
| Routine monitoring of neglected areas | Surveillance of WNV and climate data |
| Vaccination of hosts in high-risk areas | - |
| **Predictive modes of future climate change impacts** | |
| Combination of laboratory data with real data | Consider WNV vector information in predictive modeling |
| Consideration of factors affecting WNV ecology | Considering changes in WNV evolutionary trajectories |
| Higher spatial and temporal resolution of cases  and more accurate climate and mosquito data | Integration of mosquito, virus, and host data |
| Predicting monthly changes in disease patterns | Understanding the biology of virus-vector-host interactions |
| **Cross-disciplinary/border cooperation** | |
| Building cross-sectoral and interdisciplinary early warning systems based on the One Health monitoring approach | Creative new methodologies and interdisciplinary expertise |
| Integrating interdisciplinary research in human, veterinary and environmental health | Promoting cross-border cooperation |
| **Public education** | |
| Ongoing health education | Organization of educational activities |
| Enhanced awareness of personal protection | - |
| **Health system preparation** | |
| Training of health professionals to improve diagnostic techniques for WNV | Improve climate adaptation capacity |
| Improvement of basic medical and security facilities | Developing a strong plan for extreme heat and heavy precipitation |
| Rationalization of resources for disease prevention and control | - |
| **Environmental management** | |
| Elimination of reproduction and breeding sites for WNV vectors | Biodiversity conservation |
| Limiting greenhouse gas emissions & controlling warming | - |
